# Survival Behavior of *Lactobacillus plantarum* Loaded in Alginate/Konjac Glucomannan Composite Beads Under Storage and Gastrointestinal Conditions

**DOI:** 10.1002/fsn3.71322

**Published:** 2025-12-06

**Authors:** Weifeng Chen, Kunpeng Zhao, Jiaxiang Zang, Richao Hao, Bingbing Liu, Hongtao Du, Wei Xu

**Affiliations:** ^1^ School of Food and Bioengineering Henan University of Animal Husbandry and Economy Zhengzhou China; ^2^ College of Life Science Xinyang Normal University Xinyang China; ^3^ Dabie Mountain Laboratory Xinyang China

**Keywords:** bead, konjac glucomannan, *Lactobacillus plantarum*, sodium alginate, stability

## Abstract

Encapsulation techniques are commonly used to preserve the viability of probiotics during storage and digestion. In this paper, composite beads were developed using sodium alginate (ALG) and konjac glucomannan (KGM) by calcium cross‐linking, and were further used to entrap *Lactobacillus plantarum* for withstanding adverse environments. The results showed that the ALG/KGM solution displayed solid‐like behavior after KGM addition. Fourier transform infrared spectroscopy (FTIR) and X‐ray diffraction (XRD) indicated that the beads were prepared through crosslinking agent ions with the carboxylate functional groups. The encapsulation efficiency (EE) of the ALG/KGM bead with ratios of 1:3 (AK13) reached 81.5% with a loading capacity (LC) of 8.15 log CFU/g. Besides, the ALG/KGM beads enhanced the storage stability at 4°C. *L. plantarum* count continually increased with a maximum of 11.8 log CFU after 40 min gastric digestion. The work confirmed that the ALG/KGM bead was an excellent probiotic protective carrier for *L. plantarum* .

## Graphical abstract

ALG/KGM bead was used as a protective and delivery carrier for *Lactobacillus plantarum* . The beads enhanced the storage stability of *Lactobacillus plantarum* at 4°C. *Lactobacillus plantarum* count greatly increases after 40 min gastric digestion.

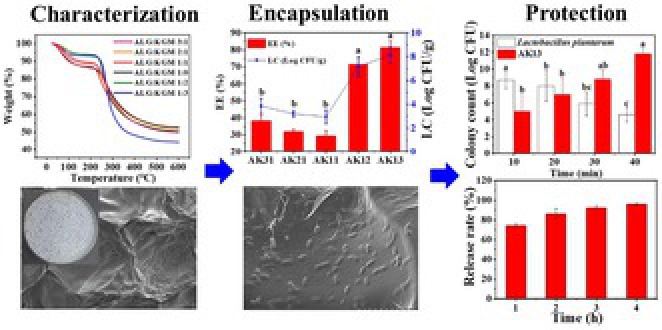

## 1 Introduction

Probiotics, as live microorganisms that exert beneficial effects in the gut after ingestion, have received extensive attention in the food and pharmaceutical industry (Mazziotta et al. 2023; Khan et al. 2023). They are usually added into foods and dietary supplements as active ingredients. Many researchers have confirmed the health benefits of *Lactobacillus plantarum* , especially in the prevention of intestinal inflammation, obesity, rectal cancer (Chen et al. 2023; Wang, Xiong, et al. 2024; Aljohani et al. 2025). However, only a small quantity of living *L. plantarum* cells enter through the intestine, and then influence the intestinal flora environment. The quantity and activity of *L. plantarum* both significantly reduce during transport, storage, processing, and delivery (AL‐Fawares et al. 2025; Sun, Wang, et al. 2024; Misra et al. 2022).

Biomacromolecule encapsulation systems are commonly prepared to improve the activity of probiotics in the gut (Thinkohkaew et al. 2024; Han et al. 2024; Wang, Huang, et al. 2024). For example, cruciferin/alginate microcapsule provided the physical barrier to improve the thermal stability and shelf life of *Limosilactobacillus reuteri* (Akbari et al. 2023). It was found that the incorporation of polymeric and other fillers could significantly enhance the stability of *Lacticaseibacillus casei* when encapsulated in calcium alginate microgels (Xing et al. 2024; Hao et al. 2024). In these encapsulated systems, sodium alginate (ALG) was a very versatile polysaccharide for producing gel beads for delivering *L. plantarum* . ALG enriched with guluronic can be cross‐linked with divalent cations to form a characteristic “egg box” structure (Ni et al. 2023; Nan et al. 2023). However, the calcium alginate gel beads commonly have several disadvantages, such as porous network structures, low encapsulation efficiency (EE), fast release behavior of core materials, and short shelf life in food systems (Sun, Gao, et al. 2024; Cortés‐Camargo et al. 2023).

Konjac glucomannan (KGM) is a natural, nonionic, water‐soluble polysaccharide, which has excellent biocompatibility and degradability (Kapoor et al. 2024; Zhang et al. 2025). It was found that KGM and other polysaccharides formed composite hydrogels with favorable film‐forming and mechanical properties (Basak and Singhal 2024; Qiao et al. 2023; Zhang and Rhim 2022). For example, the synergistic effect between KGM and wheat gluten protein can effectively improve the undesirable properties of wheat gluten protein, such as high brittleness, easy dehydration, and shrinkage (Zeng et al. 2024; Xu et al. 2025). However, limited study has been conducted on ALG/KGM composite bead loading *L. plantarum* . Therefore, the beads were developed using ALG and KGM by calcium cross‐linking, and further tailored to encapsulate and protect *L. plantarum* .

In this study, composite beads with different ratios of ALG/KGM were prepared by calcium cross‐linking, which were used to entrap *L. plantarum* withstand the challenges of an adverse environment. It was characterized by Fourier transform infrared (FTIR) spectroscopy, X‐ray diffraction (XRD) spectroscopy and scanning electron microscopy (SEM). Furthermore, the delivery performance of *L. plantarum* during environments, heat treatment, and gastrointestinal resistance was further investigated.

## 2 Materials and Methods

### 2.1 Materials

ALG (M_W_ 1.2 × 10^5^–2.0 × 10^6^ Da) was purchased from Sinopharm Chemical Reagent Co. Ltd. (China). KGM (average Mw 1.4 × 10^6^ Da, KGM ≥ 92%, ASH ≥ 0.61%, protein ≥ 0.17%) was kindly provided by Hubei Yizhi Konjac Industry Co. Ltd. *L. plantarum* was separated from pickles and identified using MALDI TOF by the Henan Key Laboratory of Unconventional Feed Resources Innovative Utilization. The MRS agar medium, NaCl, and other chemicals were reagent grade and purchased from Sinopharm Chemical Reagent Co. Ltd. (China). All the solutions in the experiments were prepared using ultrapure water through a Milli‐Q water system (Millipore, MA, USA).

### 2.2 Rheological Properties of ALG/KGM Solution

First, ALG (2 wt%) was dissolved in water by stirring for 5 h at room temperature. The same concentration of KGM was prepared and filtered using 200 mesh filter cloth. The ALG/KGM solutions were mixed with different ratios (3:1, 2:1, 1:1, 1:2, 1:3) for another 3 h. Shear rheology and frequency sweeps of the ALG/KGM solutions were performed using a DHR‐2 rheometer (TA, USA). A parallel plate geometry with a diameter of 40 mm and a gap of 1 mm was used for all shear flow and frequency tests. For shear flow tests, the shear rate increased from 0.1 to 100 s^−1^ at 25°C. Frequency sweep was performed by increasing the frequency from 0.08 to 100 Hz at strain 0.2% which was within the linear viscoelastic range.

### 2.3 Preparation of ALG/KGM Composite Beads

ALG/KGM composite beads were fabricated based on ALG gelatinization through ionic gelation using the previous method with minor modification (Jiang et al. 2024). ALG/KGM blend solutions (10 mL) with different ratios (3:1, 2:1, 1:1, 1:2, and 1:3) were loaded in a 10 mL syringe, and then dripped into calcium chloride (2 wt%) solution with a needle of 0.5 mm diameter. The distance between the needle and the CaCl_2_ solution was fixed at 15 cm, and the solution flow rate was 2.0 mL/min. Meanwhile, the CaCl_2_ solution was continually stirred at 300 rpm. The composite beads were then aged for another 20 min. Finally, the beads were collected for further experiments. The composite beads were defined shortly as AK31, AK21, AK11, AK12, and AK13, respectively.

### 2.4 Characterization of ALG/KGM Composite Beads

Before characterization, all ALG/KGM composite beads were dried using a freeze vacuum dryer (SCINTZ‐10, China). FTIR spectra of ALG/KGM composite beads were recorded using an FTIR spectrometer (PerkinElmer, USA) from 4000 to 400 cm^−1^ with 4 cm^−1^ resolution. The voltage and current of XRD (Smartlab9, Rigaku, Japan) were 45 kV and 100 mA, respectively. In addition, the scanning angle ranges from 5° to 90° with the scanning speed of 10°/min. The thermal stability of ALG/KGM composite beads (10 mg) was performed using TGA (SDTQ600, TA Instruments, USA) with the temperature increasing from 20°C to 600°C under a nitrogen atmosphere.

### 2.5 Loading Capacity and Encapsulation Efficiency of *L. plantarum*

*Lactobacillus plantarum* was inoculated on 100 mL MRS medium and incubated at 37°C for 16 h. The culture medium was then centrifuged at 8000 rpm and 4°C for 10 min. Then *L. plantarum* was dispersed and washed with 0.85% sterile saline twice. The final *L. plantarum* concentration in sterile saline was 2 × 10^10^ CFU/mL. *L. plantarum* was added into ALG/KGM composite solutions, and beads were fabricated as described in the above method. The *L. plantarum*‐loaded beads were put into a PBS solution and stirred for 30 min. After homogenization for 30 s to destroy its structure, the mixture was diluted and counted using the plate‐counting method. The loading capacity (LC) and EE were calculated using the equation below (Mu et al. 2018; Liu et al. 2023):

$$\begin{array}{c} \text{Encapsulation efficiency}\;\left(\%\right)\\ \;=\text{collected viable count}/\text{total viable count}\times 100\% \end{array}$$

$$\begin{array}{c} \text{Loading capacity}\;\left(\mathrm{CFU}/g\right)\\ \;=\text{collected viable count}/\text{mass of the gels} \end{array}$$

### 2.6 Stability of *L. plantarum* During Environment and Heat Treatment

Stability of *L. plantarum* was tested by storage and thermal treatment. Free *L. plantarum* and AK31 were stored in 10 mL sterile saline at 4°C for 6 days. The mediums were removed at 0, 2, 4, and 6 days, respectively, and the number of viable bacteria in them was measured. Then, free *L. plantarum* cells and AK31 were added and kept for 5 min. The glass bottle was then cooled in an ice bath for 10 min to measure the number of viable bacteria.

### 2.7 Gastrointestinal Resistance of *L. plantarum* Loaded Beads

The simulated gastric fluid (SGF) was prepared by NaCl (2 g), pepsin (3 g), distilled water (1 L) and then the pH was adjusted to 2.0 with HCl. Simulated intestinal fluid (SIF) was prepared by 0.65% KH_2_PO_4_, 1 g trypsin, 18 g bovine bile salt, and the pH was adjusted to 7.4 with NaOH. AK31 (2 g) was stirred at 100 rpm in 18 mL SGF at 37°C. Free *L. plantarum* was used as the control. After digestion in SGF for 0, 10, 20, 30, 40 min, respectively, the hydrogel beads were removed and added to 18 mL sodium citrate solution. The beads were cracked to release *L. plantarum* , and then diluted with sterile normal saline. The total time of digestion in SIF was 4 h. The supernatant was taken at 1, 2, 3, and 4 h, respectively. The number of viable bacteria released was measured by gradient dilution. The survival rate in the SGF and release rate (RR) in SIF is calculated according to previous reports (Wang, Ma, et al. 2024; Xie et al. 2024).

### 2.8 Micromorphology of *L. plantarum* Loaded ALG/KGM Composite Beads

*Lactobacillus plantarum* loaded ALG/KGM composite beads were lyophilized using a vacuum freeze dryer for 48 h. The lyophilized beads were sprayed with platinum, and then the surface morphology was observed using a SEM (Regulus 8220, Hitachi, Japan) at an accelerating voltage of 20 kV. The micromorphology of *L. plantarum* loaded beads after simulated digestion in vitro was characterized by Cryo‐SEM (FEI Quanta 450, USA) under high vacuum conditions.

### 2.9 Statistical Analysis

The results were expressed as mean ± standard deviation. Analysis of variance (ANOVA) and Duncan's multiple tests were conducted based on SPSS 25.0 software. The difference was significant at *p* < 0.05. Origin 9.1 software was used for data analysis.

## 3 Results and Discussion

### 3.1 Rheological Properties of ALG/KGM Solutions

**FIGURE 1:**
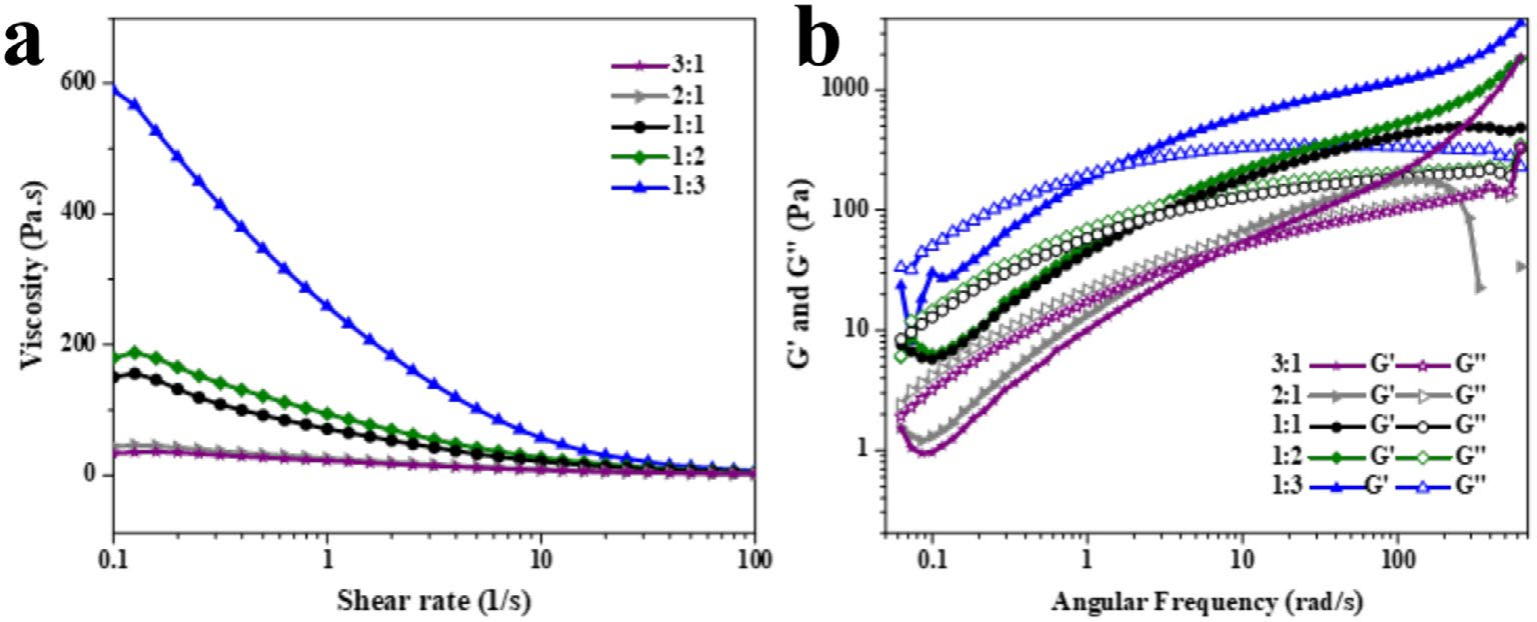
Viscosity as a function of shear rate (a) and frequency sweeps (b) of ALG/KGM solution. G′ and G″ indicate the storage modulus and loss modulus, which respectively represent the key characteristics of the system's elasticity and viscosity.

Figure 1a showed the flow shear behavior of SA/KGM solutions. The viscosity displayed an increasing trend as the KGM content increased. When KGM was added to the ALG solution, a thickening effect was noted. Compared with the ALG solution, the increase effect was determined by the ratio of ALG and KGM. This thickening effect had been reported previously. KGM is a common structural or thickening agent in food systems to improve the stability of the continuous phase (Chen et al. 2024; Li, You, et al. 2024). All solutions showed shear‐thinning and non‐Newtonian fluid behavior (Table 1). The larger the KGM concentration, the faster the apparent viscosity decreased with the increase of shear rate. The apparent viscosity of ALG varied almost linearly with the shear rate. Comparatively, the viscosity of ALG/KGM solutions exerted a more significant effect on shear rate. The phenomena could be explained by disturbing the entangled ALG/KGM network during shearing. At low shear rate, the viscosity was nearly unchanged. When the shear rate increased, there was enough time to recover the disordered state for entangled ALG/KGM.

**TABLE 1:** The fitted results of the Power‐Law model of SA/KGM solutions, *n* and *k* indicate flow index and consistency index.

*Y* = k*x ^(*n*−1)^	*k*	*n*	*R* ^2^
ALG:KGM = 3:1	2.3747 ± 0.0474	0.8109 ± 0.0112	0.93
ALG:KGM = 2:1	20.0151 ± 0.5286	0.6833 ± 0.0159	0.96
ALG:KGM = 1:1	85.3095 ± 2.2108	0.6097 ± 0.0155	0.98
ALG:KGM = 1:2	66.3832 ± 1.3991	0.5914 ± 0.0125	0.98
ALG:KGM = 1:3	23.6347 ± 0.5424	0.6576 ± 0.0138	0.97

Figure 1b showed both storage modulus (G′) and loss modulus (G″) were sensitively affected by KGM addition. The values of G′ and G″ increased with frequency for all ALG/KGM solutions. ALG solutions with or without KGM displayed the dominant liquid property at lower frequencies. While they showed solid‐like behavior at high frequencies after KGM addition. This finding agreed with the entanglement theory and the viscosity results above. Meanwhile, the shift of the crossover frequency of G′ and G″ also depended on KGM addition. The crossover frequency shifted to low frequencies when KGM content increased. The crossover frequency value was 8.05 Hz for ALG/KGM solution with a ratio of 3:1. whereas the value decreased to 1.65 Hz when the ratio was 1:3. This phenomenon showed that an enhancement of temporary ALG/KGM network structures was formed induced by intensive hydrogen bond interaction.

### 3.2 Swelling Behavior of ALG/KGM Composite Beads

**FIGURE 2:**
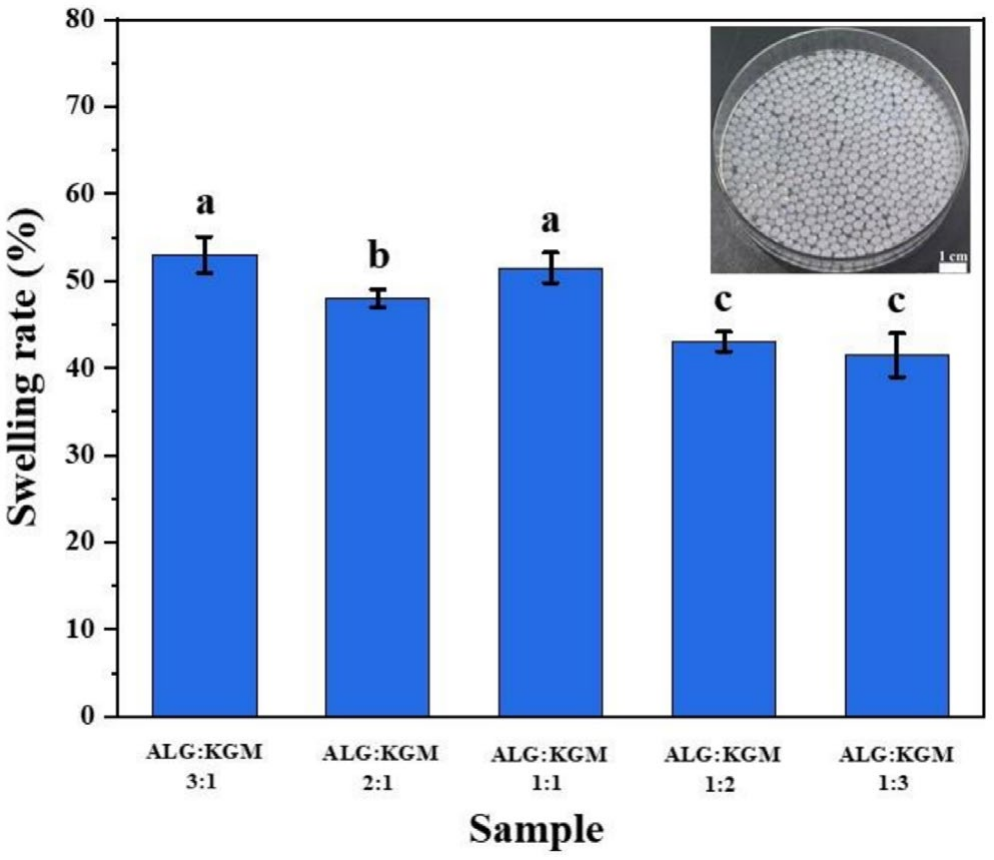
Swelling behavior of ALG/KGM composite beads. Different alphabets in the same column demonstrate significant differences (*p* < 0.05).

The swelling behavior of ALG/KGM beads at pH 7.4 was shown in Figure 2. It showed that ALG beads with or without KGM displayed a decreased swelling behavior in the medium. It was noted that the swelling ratio of ALG beads was 69% ± 2%. While the swelling ratio decreased in the presence of KGM. Meanwhile, it continued to decrease as the ratio of ALG and KGM decreased. The decreased swelling behavior may result from the corrosion behavior of ALG/KGM beads. The corrosion behavior of ALG could be reduced through calcium ion crosslinking. While the good water solubility of KGM could further increase the dissolution in water. Even so, all the ALG/KGM beads maintained complete structure after 24 h. The less exchange with external solutions may provide potential protective behavior for *L. plantarum* .

### 3.3 Characterization of ALG/KGM Composite Beads

**FIGURE 3:**
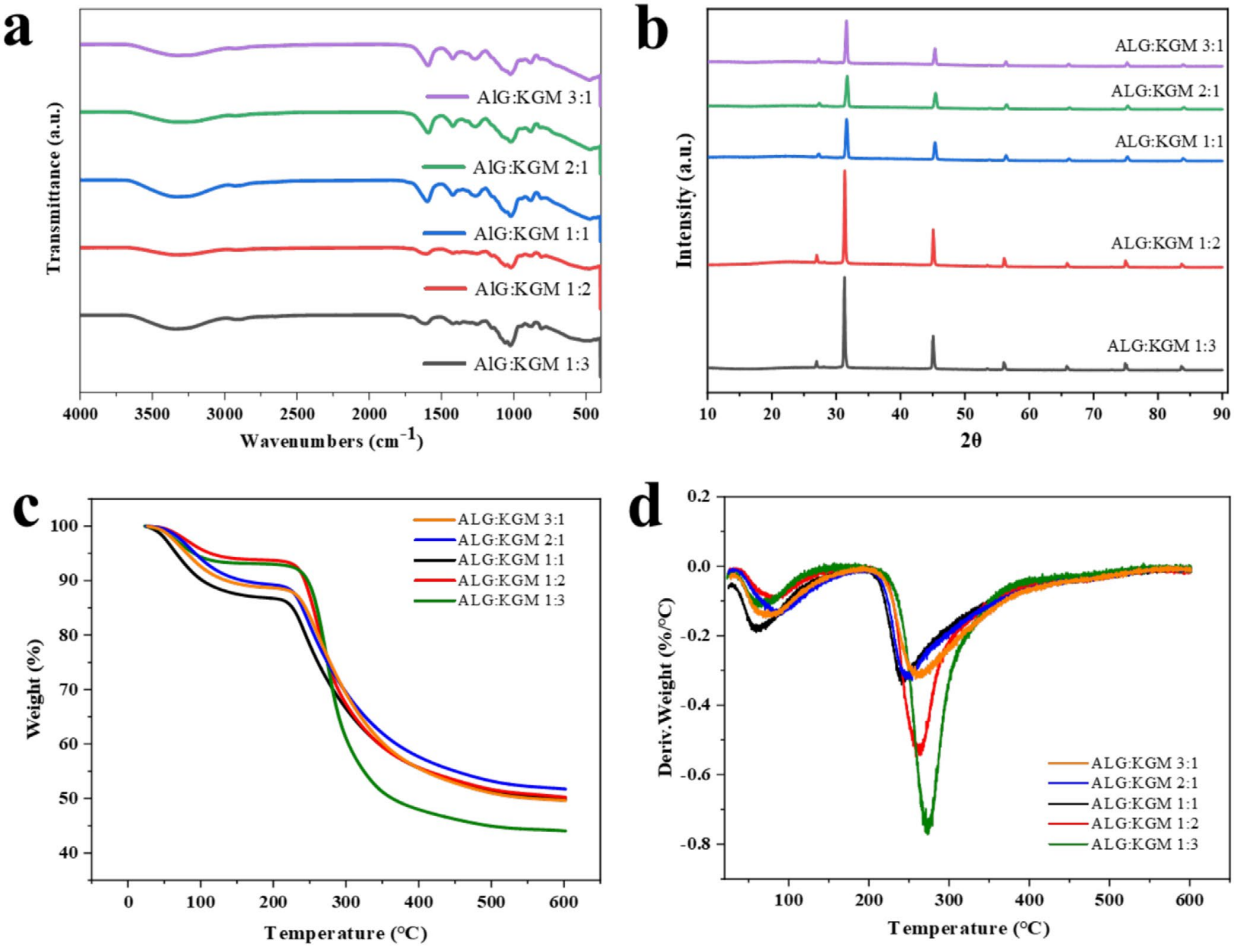
Fourier transform infrared spectroscopy (FTIR) (a), X‐ray diffraction (XRD) (b), thermogravimetric analysis (TG) (c) and derivative thermogravimetric (DTG) (d) of ALG/KGM composite beads. FTIR investigated the variation of chemical groups and intermolecular interactions. XRD determined the crystal structure of ALG and KGM during bead formation. TG and DTG curves highlighted the rate of weight loss and identified distinct thermal degradation stages.

FTIR spectroscopy is commonly employed to investigate the variation of chemical groups in composites. In order to explore the interaction between KGM and ALG, FTIR of composite beads prepared from different ratios of ALG/KGM was measured. In Figure 3a, ALG beads showed that the absorption band around 2950, 1620, and 1040 cm^−1^ corresponds to the stretching of ‐CH, ‐COOH, and C‐O‐C, respectively. For ALG/KGM beads, the peaks of ALG beads almost appeared in the spectroscopy. Although some peaks became weak due to interaction or superposition between groups of ALG and KGM. The results indicated that ALG/KGM beads have no chemical reaction between KGM and ALG, which contain both molecular characterizations. The XRD results were well agreed with the phenomenon of FTIR. It is noted that all ALG/KGM beads exhibited remarkable peaks at positions 32.16 and 45.83 that resembled an amorphous structure. However, the diffraction peaks of the beads shifted depending on the ratios of ALG and KGM which indicated the fabrication of beads based on the crosslinking agent ions with the carboxylate functional groups. The peak intensities were dependent on the added polymer concentration.

Figure 3c showed the TG thermograms and derivative of TG (DTG) of ALG/KGM composite beads. It noted that all beads had a small mass loss in the early stage from 30°C to 100°C. The first mass loss was reduced by water evaporation. At the stage of temperature from 100°C to 400°C, the obvious mass loss was observed for all beads. The retained mass reached 57.6% for composite ALG/KGM beads as the ratio was 2:1. while the retained masses reached 55.2% and 47.5% as the ratios were 1:1 and 1:3, respectively. The results showed that the thermal stability of ALG/KGM composite beads decreased as KGM increased. The maximum weight loss points of composite beads were at 260°C and 280°C as the ratios of ALG and KGM were 3:1 and 1:3, respectively. According to a previous study, the thermal stability of composite beads was mainly determined by ALG content and gelation force during composite beads fabrication, especially crosslinking with Ca^2+^ (Zheng et al. 2024; Gao et al. 2024).

### 3.4 Encapsulation of *L. plantarum* Based on ALG/KGM Composite Beads

**FIGURE 4:**
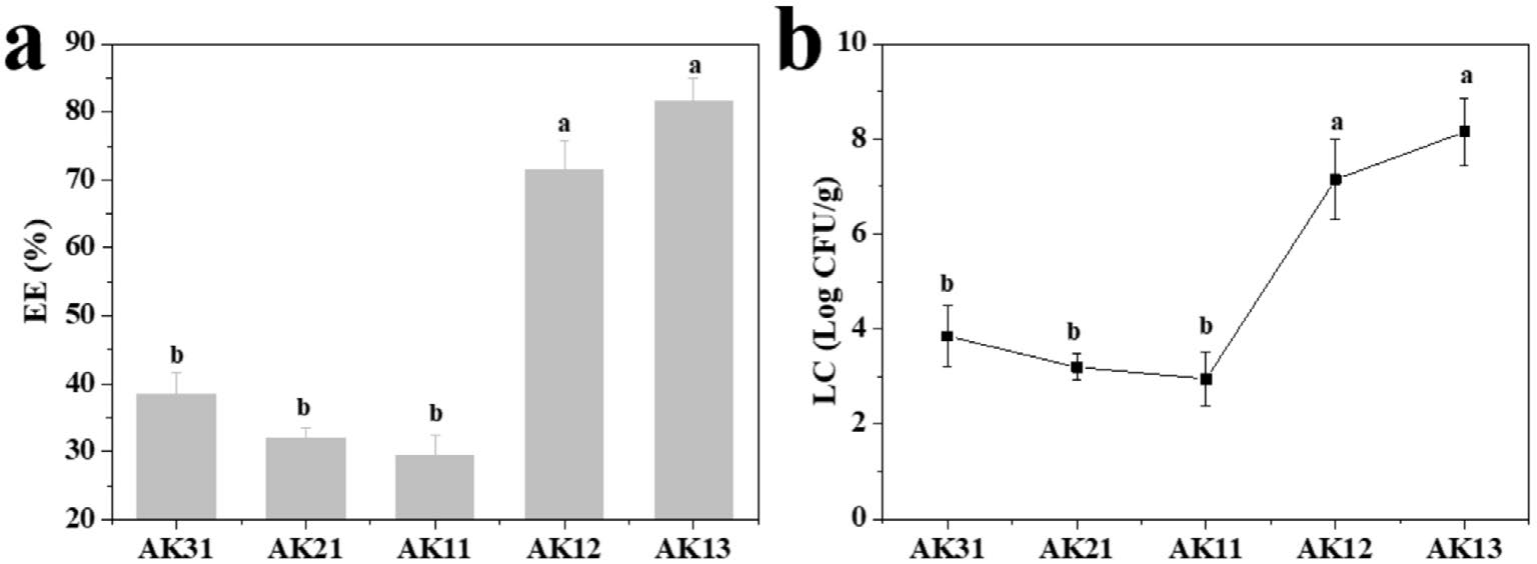
Encapsulation efficiency (a) and loading capacity (b) of *Lactobacillus plantarum* based on ALG/KGM composite beads. Different alphabets in the same column demonstrate significant differences (*p* < 0.05).

It noted that the EE and LC shared a similar trend and were significantly regulated by the constituent of composite beads (Figure 4). The EE and LC of AK31 were 33.87% and 3.85 log CFU/g. Low‐dose KGM addition made the EE of LC reduce. As the ratio of ALG and KGM was 1:1, the EE and LC reached the minimum values of 29.5% and 2.95 log CFU/g, respectively. While EE and LC sharply increased as KGM continually increased. The EE of AK13 reached 81.5% with a maximum LC of 8.15 log CFU/g. It showed that the great addition of KGM greatly enhanced the EE and LC of ALG/KGM composite beads. The result was well agreed with previous studies (Wang, Zhang, et al. 2024; Phumsombat et al. 2024). This was attributed to the fact that ALG beads often have large pores and low EE. Therefore, it is typically combined with other macromolecules to enhance the embedding efficiency. On the other hand, KGM possesses favorable film‐forming characteristics. The dense shell serves to protect the core and improve the EE, thereby preventing the premature release of probiotics in the solution.

### 3.5 Microstructure of *L. plantarum* Based on ALG/KGM Composite Beads

**FIGURE 5:**
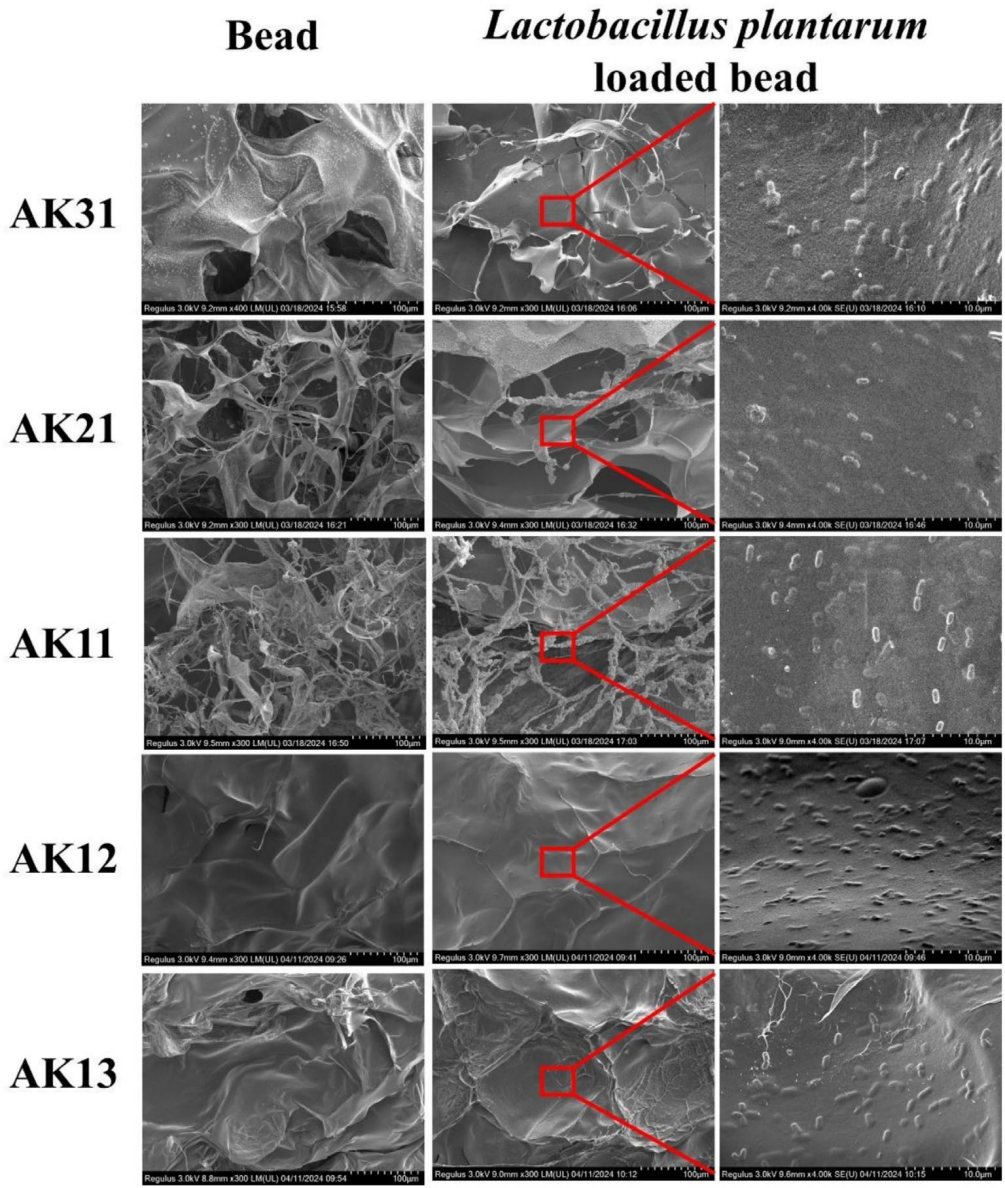
Scanning electron microscopy images of ALG/KGM composite beads and *Lactobacillus plantarum‐loaded* ALG/KGM composite beads.

The microstructure of ALG/KGM beads was observed in Figure 5. It revealed the presence of large pores on the surface for AK31. The porous structure facilitated the diffusion of the beads. While the surface trended to be smoother and flatter with KGM addition. The phenomenon might result from the film‐forming property of KGM, which enhanced the release ability and decreased the EE and LC (Meng et al. 2022). The *L. plantarum* loading nearly did not affect the structure of ALG/KGM composite beads which illustrated that the *L. plantarum* was filled in the pores of the gel. The surface of all beads presented a large number of *L. plantarum* . Compared with AK12 and AK13, nearly all *L. plantarum* were successfully encapsulated in the beads' internal region. The results well agreed with the above results (Figure 4).

### 3.6 *Lactobacillus plantarum* Stability During Storage and Heat Treatment

**FIGURE 6:**
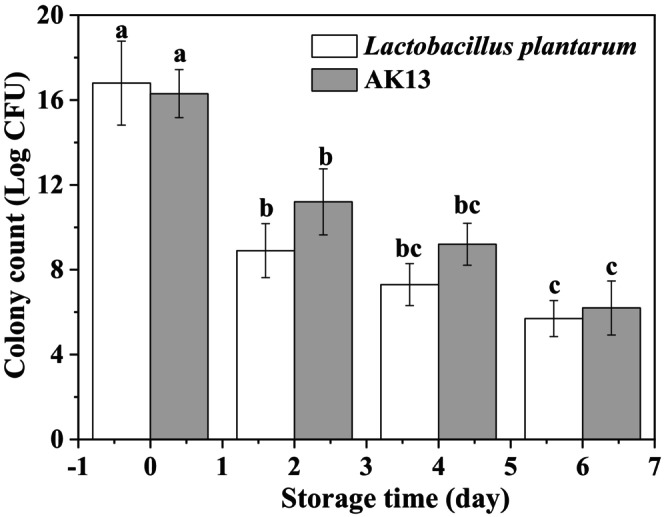
The stability of *Lactobacillus plantarum* during storage with and without bead loading. Different alphabets in the same column demonstrate statistically significant differences (*p* < 0.05).

Storage and thermal stability are crucial characteristics for evaluating probiotic products. The stability of the *L. plantarum* and ALG/KGM beads entrained *L. plantarum* during storage at 4°C was observed in Figure 6. Whether packaged or not, *L. plantarum* counts in both conditions decreased during storage time. It was noted that *L. plantarum* counts without bead protection reached 5.7 log CFU, which reduced to 67% compared with the primary value when *L. plantarum* was stored for 6 days at 4°C. While the counts in ALG/KGM composite beads decreased by only 10 log CFU. This demonstrated that the bead endowed *L. plantarum* with superior storage stability. This suggested that the embedding effect of ALG/KGM beads enhanced the storage stability of *L. plantarum* in a low‐temperature environment. The possible reason for this phenomenon was that ALG and KGM, acting as prebiotics, facilitated the growth of *L. plantarum* . Additionally, the core‐shell bead structure aided the internal *L. plantarum* in withstanding adverse conditions.

### 3.7 Stable of *L. plantarum* During in Vitro Gastrointestinal Digestion

**FIGURE 7:**
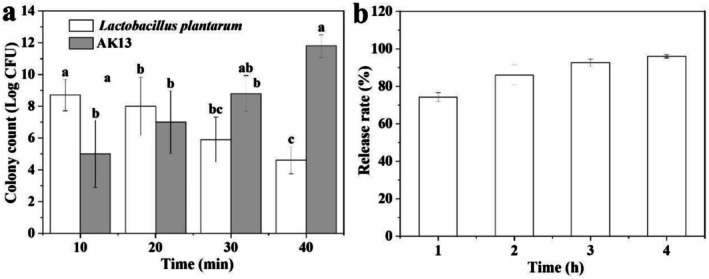
Survival rate of *Lactobacillus plantarum* in simulated gastric fluid (a) and release behavior (b) in simulated intestinal fluid. Different alphabets in the same column demonstrate significant differences (*p* < 0.05).

The acid tolerance of free *L. plantarum* and AK13 was compared studied in SGF. In the SGF environment (pH 2.0), *L. plantarum* count greatly reduced to 8 log CFU after 20 min, and 4.6 log CFU/mL after 40 min of incubation which was only 53% of the original *L. plantarum* concentration (Figure 7). The results indicated that *L. plantarum* was sensitive to the extreme sour environment. It was unfavorable to its growth. The phenomenon was also confirmed in other research work (Li, Jiang, et al. 2024; Misra et al. 2025). While it was noted that *L. plantarum* counts in AK13 increased with different degrees after incubation time. *L. plantarum* count greatly increased to 7 log CFU after 20 min of incubation, and the value continually increased with a maximum of 11.8 log CFU (2.36 times) after 40 min of incubation. Compared to the original *L. plantarum* count, the value increased more than double. The phenomenon may result from the bead providing a protective structure from the acid tolerance. Meanwhile, the prebiotic ALG and KGM were helpful to the growth of *L. plantarum* .

Intestinal fluid (SIF) could provide a more direct assessment of survival rate and release behavior of *L. plantarum* after ALG/KGM beads loading. In the continuous gastrointestinal simulation experiment, the RR of *L. plantarum* after 1 h incubation was 74% for AK13. While the rate was found to be 96% when incubated 4 h in SIF. It was reported that the RR of probiotics was 88.16% after 5 h reaction in chitosan embedded ALG/ *Lycium barbarum* polysaccharide (LBP) gels (Liu et al. 2024). Comparatively, the RR was greatly improved for ALG/KGM beads. The reason for this phenomenon is that H^+^ in SGF reacted with ‐COO in ALG, which changed the “egg‐box” structure and made it more likely to swell as it was transferred to the SIF. On the other hand, the high water solubility of KGM contributed to the release behavior of *L. plantarum* .

### 3.8 Cryo‐SEM Observation of *L. plantarum* Loaded Beads After Gastrointestinal Digestion

**FIGURE 8:**
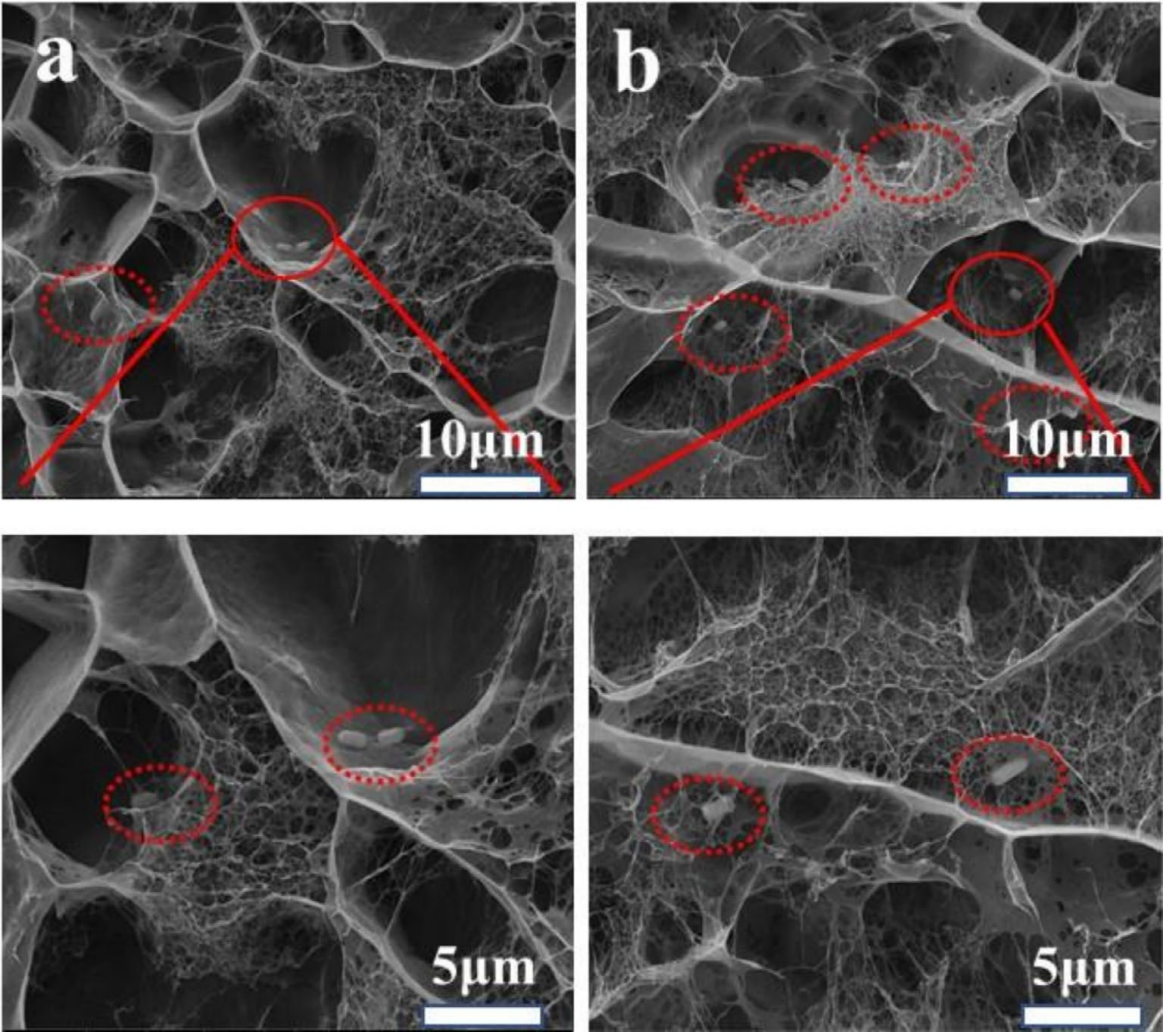
Cryo‐SEM observation of *Lactobacillus plantarum* before and after gastric digestion.

To observe a more accurate spatial structure, Cryo‐SEM was used to in situ investigate the microstructure of *L. plantarum* loaded beads after gastrointestinal digestion (Figure 8). Whether gastrointestinal digestion occurred or not, AK13 displayed heterogeneous microstructures with coexistence of intertwined fibrous aggregation and networks, which mainly resulted from the crosslinking between ALG/KGM and Ca^2+^. The structure was well agreed with the above SEM results. Before gastrointestinal digestion, the backbone fraction of *L. plantarum* beads was large with tightly distributed pores. The gaps between the backbones were abundant and appeared as interpenetrating networks. While the backbones shrank with much more fibrous aggregates distributing in the cavities. The comparative change could be clearly displayed at higher magnifications. In particular, *L. plantarum* count seemed to increase after simulated gastric digestion. The phenomenon illustrated that *L. plantarum* cells could grow during the environment of gastric digestion. The Cryo‐SEM observation further confirmed that the ALG/KGM composite beads could provide not only a protective structure, but also a nutritional environment for *L. plantarum* . The results were consistent with the previous result of Figure 7.

## 4 Conclusion

In this study, ALG/KGM composite beads with different ratios were innovatively prepared by calcium cross‐linking, which were used to entrap *L. plantarum* withstanding the challenges of adverse environments. ALG/KGM solution showed an increasing trend in viscosity and solid‐like behavior at high frequencies. The swelling ratio of composite beads decreased in the presence of KGM. EE of AK13 reached 81.5% with a maximum LC of 8.15 log CFU/g. Composite beads enhanced the storage stability of *L. plantarum* at 4°C. *L. plantarum* count greatly increased and reached a maximum of 11.8 log CFU after gastric digestion. The ALG/KGM composite beads exhibited a protective structure and nutritional environment for *L. plantarum* , which could be applied in intestinal function ingredients and food.

## Author Contributions

**Weifeng Chen:** methodology, writing – review editing. **Kunpeng Zhao:** data curation. **Jiaxiang Zang:** validation. **Richao Hao:** data curation. **Bingbing Liu:** investigation, validation. **Hongtao Du:** writing – review editing. **Wei Xu:** supervision, project administration.

## Funding

Research grants from the Program for Science and Technology Innovation Talents in Universities of Henan Province (Grant No. 24HASTIT060), the Natural Science Foundation of Henan Province (Grant No. 252300420218), the Project of Henan Provincial Science and Technology (Grant No. 222102310438), University‐Level Young Backbone Teacher of Henan University of Animal Husbandry and Economy (C3030614), and National Natural Science Foundation of China (Grant No. U2004160).

## Ethics Statement

The authors have nothing to report.

## Conflicts of Interest

The authors declare no conflicts of interest.

## Data Availability

The data that support the findings of this study are available from the corresponding author upon reasonable request.
